# Postpartum anemia and maternal wellbeing: A cohort analysis of the WOMAN‐2 trial

**DOI:** 10.1002/ijgo.70969

**Published:** 2026-04-03

**Authors:** The WOMAN‐2 Trial Collaborators, Judith Lieber, Katharine Ker, Folasade Adenike Bello, Rizwana Chaudhri, Kiran Javaid, Aasia Kayani, Mwansa Ketty Lubeya, Projestine Muganyizi, Oladapo Olayemi, Bellington Vwalika, Monica Arribas, Eni Balogun, Amy Brenner, Amber Geer, Poppy Mallinson, Raoul Mansukhani, Danielle Prowse, Haleema Shakur‐Still, Ian Roberts, France Donnay, Racheal Phiri, Tina Lavender, Ghazala Mahmud, Haleema Shakur‐Still, Ian Roberts, Jens Kieckbusch, James Lawrence, Sari Watson, Jane Armitage, Maria Quigley, Olufemi T. Oladapo, Sabaratnam Arulkumaran, Imelda Bates, Rizwana Chaudhri, Bukola Fawole, Katharine Ker, Ian Roberts, Haleema Shakur‐Still, Eni Balogun, Danielle Prowse, Danielle Beaumont, Charlotte Fleming, Monica Arribas, Collette Barrow, Llion Roberts, Amber Geer, Julio Gil Onandia, Madeleine Cargill, Andrew Thayne, Christiana Alao, Petula Buck, Raoul Mansukhani, Tim Collier, Katharine Ker, Mbwana Mohammed, Amy Brenner, Myriam Benyahia, Judith Lieber, Haleema Shakur‐Still, Ian Roberts, Rizwana Chaudhri, Aasia Kayani, Kiran Javaid, Projestine Muganyizi, Alice Kawala, Rose Temba, Oladapo Olayemi, Folasade Adenike Bello, Olujide Okunade, Bellington Vwalika, Aziz‐un‐Nisa Abbasi, Ruqqia Sultana, Shehla Noor, Ansa Islam, Sadia Bibi, Sundus Khan, Wajeeha Khurshid, Shahida Magsi, Basma Zia, Haleema Yasmin, Khadija Bano, Saba Khan, Naheed Fatima, Bushra Sher Zaman, Sohail Mahmood Chaudhry, Shakila Yasmin, Khiaynat Sarwar Hashmi, Nadia Babar, Arif Tajammul, Alia Bashir, Farah Siddique, Fouzia Perveen, Nusrat Shah Kamal, Riffat Jaleel, Sarah Kazi, Aruna Hira, Pushpa Makhija, Syeda Batool Mazhar, Nasira Tasnim, Saera Afghan, Mussarat Batool, Sobia Luqman, Fouzia Kashif, Shaista Hifaz Abro, Javeria Abro, Najma Ghaffar, Rakhshanda Mushtaq, Safia Bibi, Shamsa Humayun, Zohra Khanum, Noreen Akmal, Shamila Ijaz Munir, Aisha Malik, Amna Zia Eusaph, Sofia Manzoor, Khola Yousaf, Misbah Kausar, Yasmeen Waqas, Naila Ehsan, Aisha Siddiqa, Uzma Suhail Afridi, Nosheen Sikandar, Rehana Kamaal, Mehnaz Khakwani, Rashida Perveen, Huma Quddusi, Hajira Masood, Syeda Ali, Asma Akhter, Mubashra Samina, Sana Ishfaque, Rizwana Chaudhri, Humaira Bilqis, Shahid Irshad Rao, Saima Ashraf, Uzma Shaheen, Tayyiba Wasim, Natasha Rasool, Nabeela Waheed, Khansa Iqbal, Shahida Husain Tarar, Rizwana Khalid, Rubina Sohail, Shehla Kanwal, Shagufta Saeed Sial, Humera Noreen, Naila Israr, Shehla Baqai, Asifa Siraj, Luzango Maembe, Julius Nyambarino, Enid Simon Chiwanga, Lazaro Y. Madeha, Francis Ngimwichi Joseph, Evelyne Ludovick, Baya H. Kissiwa, Sangalala Mtingele, Vincent Tarimo, Alfred Secha, Hudson August Manyanga, Idrisa Hussein, Alphonce Moyo, Delila Msigwa, France John Rwegoshora, Delfina Mkenda, Mojisola Mobolaji‐Ojibara, Michael Babalola, Owa Olorunfemi Oludele, Samuel Ojo, Oladipo Aremu, Kehinde Ogundairo, Oyewole Tunde Aremu, Gazaal Rasheedat, Iman Usman, Auwal Shuaibu, Adewale Adeyemi, Olufemi Aworinde, Theresa Irinyenikan, Mwansa Ketty Lubeya, Kenneth Chanda

**Affiliations:** ^1^ London School of Hygiene & Tropical Medicine London UK

**Keywords:** fatigue, maternal wellbeing, mental health, physical capacity, postpartum anemia

## Abstract

**Objective:**

We examined the association between postpartum anemia and maternal wellbeing in women who gave birth with anemia.

**Methods:**

We conducted a cohort analysis using secondary data from the WOMAN‐2 trial. Between August 2019 and September 2023, women with moderate or severe anemia who were giving birth vaginally were recruited from hospitals in Nigeria, Pakistan, Tanzania, and Zambia. Our exposure was postpartum anemia (hemoglobin concentration <70, 70–99, or ≥100 g/L: severe, mild/moderate, and no postpartum anemia, respectively). Our primary outcome was physical capacity (6‐min walk test). Our secondary outcomes were patient‐reported vigor, fatigue (general, physical, emotional, mental), overall fatigue, other anemia symptoms, breastfeeding difficulties, expected difficulties with usual activities, breathlessness, illness, and pain. Hemoglobin was measured at 24‐h after birth or discharge and outcomes were measured at discharge or 42 days postpartum, whichever happened first. We assessed the association between postpartum anemia and maternal wellbeing with multivariable regression models.

**Results:**

Among 15 068 participants, 11% had severe, 75% had mild/moderate, and 14% had no postpartum anemia. After adjusting for potential confounders, each 10 g/L increase in hemoglobin was associated with 2.99 (95% confidence interval [CI]: 2.12–3.86) more meters walked in 6 min. Compared to mild/moderate postpartum anemia, severe anemia was associated with expected difficulties doing usual activities (adjusted odds ratio [aOR] =1.48, 95% CI: 1.14–1.91) and other adverse wellbeing outcomes. No postpartum anemia was associated with less illness (aOR = 0.58, 95% CI: 0.35–0.96) and some adverse wellbeing outcomes.

**Conclusion:**

Low postpartum hemoglobin was associated with worse wellbeing of new mothers. Our results support recommendations to prevent and treat low postpartum hemoglobin.

## 1 INTRODUCTION

Anemia can affect over 50% of postpartum women in low‐and‐middle‐income settings and up to 50% of postpartum women in high‐income settings.^1^, ^2^ Antepartum anemia and blood loss during childbirth are the main causes of postpartum anemia. The burden of postpartum anemia is greater in low‐and‐middle‐income settings because antepartum anemia is both more common and severe. In South Asia and west and central Africa, a quarter of pregnant women have moderate or severe anemia and another quarter are mildly anemic.^3^

Anemia reduces the oxygen carrying capacity of the blood, which can impair tissue function.^4^, ^5^ Postpartum anemia is associated with adverse maternal health and wellbeing outcomes such as fatigue, reduced physical capacity, postpartum depression, and mortality.^6^, ^7^, ^8^, ^9^ Postpartum anemia may also reduce mothers' ability to bond with and care for their infant.^10^

Despite its importance in low‐and‐middle income settings, evidence on the impact of postpartum anemia is largely from small, single‐center studies in high‐income settings.^6^, ^7^, ^8^ In the present study, we examined the association between postpartum anemia and maternal wellbeing in a large cohort of women who gave birth with moderate or severe anemia in Nigeria, Tanzania, Pakistan, and Zambia.

## 2 MATERIALS AND METHODS

### 2.1 Study design and participants

We conducted a cohort (observational) analysis using secondary data from the multicenter World Maternal Antifibrinolytic Trial‐2 (WOMAN‐2 trial). Between August 2019 and September 2023, women in active labor were recruited to the WOMAN‐2 trial from 34 hospitals in Nigeria, Pakistan, Tanzania, and Zambia. Participating facilities were largely tertiary or regional referral public hospitals in provincial capitals. Hemoglobin concentration was measured using a standard point‐of‐care assessment (HemoCue Hb 201+ System) on women's arrival at hospital^11^ Women with moderate or severe antepartum anemia who were giving birth vaginally were eligible to take part. The WOMAN‐2 trial followed WHO guidelines to define moderate (hemoglobin concentration between 70 and 99 g/L) and severe (hemoglobin concentration below 70 g/L) antepartum anemia.^12^, ^13^ Women who were under 18 and lacked consent from a guardian, had a known allergy to the trial treatment, had an indication or contraindication to the intervention, or were diagnosed with postpartum hemorrhage before the umbilical cord was clamped were not eligible to take part in the trial. We have no further exclusion criteria for this cohort analysis. All women who met the WOMAN‐2 inclusion criteria were eligible to be included. The WOMAN‐2 trial protocol and results are published elsewhere.^14^, ^15^

### 2.2 Exposure and outcome measures

The exposure for this cohort analysis was postpartum anemia, defined as postpartum hemoglobin concentration <70, 70–99, or ≥100 g/L (severe, mild/moderate and no postpartum anemia, respectively).^16^ Hemoglobin was measured with a HemoCue 24‐h after birth or at discharge, whichever happened first. We used this to classify women into the exposure groups. In other words, women were not randomized to the exposure. There are no established thresholds for postpartum anemia.^17^ We used British Society of Hematology guidelines as they provide cutoffs for the first 48‐h postpartum.^16^, ^17^

Our primary outcome was physical capacity, which we assessed with the 6‐min walk test.^13^ This is an objective and validated measure of physical capacity that jointly assesses cardiovascular, neuromuscular, and other physiological systems.^13^ Women were asked to walk between two markers as many times as possible in 6‐min. The distance covered during this period was recorded by a member of the research team. Women could decline to participate or be excluded from the walk if they were not deemed well enough to attempt it.

Our secondary outcomes were patient‐reported vigor, fatigue (general, physical, emotional, mental), overall fatigue, other anemia symptoms, breastfeeding difficulties, expected difficulties with usual activities, post‐walk breathlessness, illness, and pain (Table S1). The 6‐min walk test and outcome questionnaire were done immediately before discharge. If women were still admitted to hospital on day 42, these outcomes were assessed then. Physical capacity and vigor represent “good” maternal wellbeing, while the remaining outcomes represent “poor” maternal wellbeing.

We measured vigor and fatigue (general, physical, emotional, mental) with subscales of the multidimensional fatigue symptom inventory: Short form (MFSI‐SF). This was adapted for postpartum women.^18^ The vigor subscale assesses positive feelings (e.g., “Taking into account everything since giving birth until now, I feel happy”) (Table S1). The general fatigue subscale assesses tiredness (e.g., “Since giving birth until now, I feel physically and mentally tired”). The physical fatigue subscale assesses body and muscle weakness (e.g., “Since giving birth until now, my legs feel weak”). The mental fatigue subscale assesses cognition (e.g., “Taking into account everything since giving birth until now, my mind is wandering around”). The emotional fatigue subscale assesses negative emotions (e.g., “Taking into account everything since giving birth until now, I feel depressed”). We calculated each score by summing the subscale's six Likert‐style questions (range: 0–24).^18^ We measured overall fatigue by subtracting the vigor score from the sum of the general, physical, mental, and emotional fatigue scores (range: −24–94).^18^ We dichotomized this score (≥1 representing overall fatigue).

The remaining patient‐reported outcomes were developed for, and validated in, women with postpartum anemia.^18^ We measured other anemia symptoms by summing the relevant seven Likert‐style questions (range 0–28) (Table S1). For breastfeeding difficulties, expected difficulties with usual activities, post‐walk breathlessness, illness, and pain, women were defined as having the outcome if they responded “quite a bit” or “extremely” to the relevant Likert‐style question (Table S1).

### 2.3 Statistical analysis

We developed a directed acyclic graph to identify potential confounders (Figure S1). We examined the association between postpartum anemia and physical capacity with linear regression, adjusting for potential confounders. These included sociodemographics, pregnancy history, maternal complications, and birth and baby characteristics (Table S2 for a full list of covariates). We also adjusted for time at outcome assessment. We did not adjust for surgical and non‐surgical treatments for bleeding (Figure S1) because they were closely correlated with clinical diagnosis of postpartum hemorrhage. We used negative binomial regression to model the count outcomes (fatigue subscales and other anemia symptoms) and logistic regression to model the binary outcomes. We included a random‐effect at the hospital‐level in all models to account for correlations between women attending the same hospital. Women who died were categorized as having the “worst” observation for each outcome. Women who had not yet breastfed were categorized as having “extreme” difficulties with breastfeeding. We excluded women who had a baby in the neonatal intensive care unit or perinatal death from analyses of breastfeeding difficulties.

We undertook three sensitivity analyses. We modeled the count measures as binary outcomes using logistic regression. Women were defined as having vigor, fatigue (general, physical, emotional, mental), or other anemia symptoms if they responded “quite a bit” or “extremely” to any of the relevant Likert‐style questions (Table S1). We further assessed whether postpartum anemia was associated with maternal wellbeing via time at discharge (Figure S1) by removing time at outcome assessment from our multivariable models. Finally, we repeated our multivariable models after excluding women who received a blood transfusion.

We used restricted cubic splines (4 knots) to explore the shape of the relationship between postpartum hemoglobin concentration and each outcome. We present this as adjusted predictions of physical capacity or score measures (or risks for the binary outcomes) at different concentrations of postpartum hemoglobin. We did a complete‐case analysis as there were little missing data.

We used STATA 18 and R (version 4.2.0) for our analyses.

### 2.4 Ethics

The WOMAN‐2 protocol was approved by the National Health Research Ethics Committee of Nigeria (NHREC/01/01/2007‐29/09/2019); the National Bioethics Committee of Pakistan (NBC‐340); the National Institute of Medical Research of Tanzania (NIMR/HQ/R.8a/Vol.IX/3767); the University of Zambia Biomedical Research Ethics Committee (REF 001‐04‐19); and the London School of Hygiene and Tropical Medicine's Ethics Committee (15194). A full description of consent processes is published elsewhere.^14^, ^15^

## 3 RESULTS

A total of 15 068 women took part in the WOMAN‐2 trial. There were missing data for postpartum anemia (*N* = 205), time to outcome assessment (*N* = 258), 6‐min walk test and post‐walk breathlessness (*N* = 580, respectively), breastfeeding difficulties (*N* = 287), expected difficulties with usual activities (*N* = 245), illness and pain (*N* = 240, respectively), labor duration (*N* = 85), small vulnerable newborn (*N* = 32), estimated blood loss (*N* = 10), perinatal death (*N* = 6), maternal death, clinical diagnosis of postpartum hemorrhage, and parity (*N* = 2, respectively). Table S3 shows the characteristics of women missing postpartum anemia data. There were complete data for the other variables of interest.

In the cohort, 11% of women had severe postpartum anemia, 75% had mild/moderate postpartum anemia, and 14% were not anemic after birth, 7% were diagnosed with a postpartum hemorrhage, and 25% received a blood transfusion (Table 1). Mean postpartum hemoglobin concentration was 85.8 g/L (standard deviation [SD] 13.6). A total of 18 women died. Table S3 shows the participants' characteristics by postpartum anemia status. Severe pre‐birth anemia was least common in women who were not anemic after birth (5% compared to 11% and 46% in women with mild/moderate and severe postpartum anemia, respectively). Blood transfusion was most common in women who were not anemic after birth (39% compared to 30% and 22% in women with severe and mild/moderate postpartum anemia, respectively).

**TABLE 1:** Characteristics of the participants.

	*N* (%)	Total *N*
	*N* = 15 068	15 068
Postpartum hemoglobin (g/L) (mean, SD)	85.78 (13.61)	14 863
Postpartum anemia		14 863
No anemia (≥100 g/L)	2097 (14.11%)	
Mild–moderate (70–99 g/L)	11 107 (74.73%)	
Severe (<70 g/L)	1659 (11.16%)	
Maternal age (years) (mean, SD)	27.20 (5.61)	15 068
Maternal age (years)		15 068
<20	895 (5.94%)	
20–29	8937 (59.31%)	
30–39	4830 (32.05%)	
40+	406 (2.69%)	
Country		15 068
Nigeria	1326 (8.80%)	
Pakistan	11 025 (73.17%)	
Tanzania	2029 (13.47%)	
Zambia	688 (4.57%)	
Hemoglobin at baseline (g/L) (mean, SD)	82.74 (11.84)	15 068
Anemia severity at baseline		15 068
Moderate (70–99 g/L)	12 989 (86.20%)	
Severe (<70 g/L)	2079 (13.80%)	
Multiple gestation	583 (3.87%)	15 068
Gestational age (weeks) (mean, SD)	37.39 (2.68)	15 068
Primiparity	5031 (33.39%)	15 066
Long‐term infection	552 (3.66%)	15 068
Placental abnormalities	469 (3.11%)	15 068
Hypertensive disorder	1146 (7.61%)	15 068
Small vulnerable newborn	5205 (34.62%)	15 036
Labor induction	1831 (12.15%)	15 068
Labor augmentation	4493 (29.82%)	15 068
Instrumental delivery	422 (2.80%)	15 068
Episiotomy	4355 (28.90%)	15 068
Pharmacological pain relief	1810 (12.01%)	15 068
Duration of labor (hours) (median, IQR)	9.18 (6.42–13.75)	14 983
Injury to the birth canal	2190 (14.53%)	15 068
Estimated blood loss (ml) (mean, SD)	310.31 (192.72)	15 058
Clinically diagnosed PPH	1034 (6.86%)	15 066
Treatment for PPH	485 (3.22%)	15 066
Postpartum blood transfusion	3822 (25.37%)	15 068
Postpartum intravenous fluids	4764 (31.62%)	15 068
Baby admitted to NICU	193 (1.28%)	15 068
Perinatal death before discharge	1172 (7.78%)	15 062
Small vulnerable newborn	5205 (34.62%)	15 036
Maternal death	18 (0.12%)	15 066
Time to outcome assessment (hours) (median, IQR)	12.34 (6.19–23.92)	15 019

Abbreviations: IQR, interquartile range; NICU, neonatal intensive care unit; PPH, postpartum hemorrhage; SD, standard deviation.

Hemoglobin concentration was mostly measured on the first postpartum day (median 12.6 h, interquartile range [IQR] 6.5–23.6), as were the wellbeing outcomes (median 12.3 h, IQR 6.2–23.9). On average, women walked 212.2 m during the 6‐min walk test (SD 92.8) (Table S4). The most common patient‐reported outcomes were vigor (75.7%), overall fatigue (26.2%), and difficulties breastfeeding (9.9%), and least common were illness (1.4%), pain (1.3%), and post‐walk breathlessness (0.9%) (Table S4).

Table 2 shows the adjusted associations between postpartum anemia and wellbeing outcomes (Table S5 for unadjusted results). Compared to mild/moderate postpartum anemia, severe anemia was associated with lower physical capacity (adjusted β‐coefficient −3.56 meters [m], 95% CI: −7.17–0.04) and no postpartum anemia was associated with greater physical capacity (6.37 m, 95% CI: 3.28–9.46). We modeled hemoglobin concentration as a continuous exposure for physical capacity and post‐walk breathlessness because the flexible spline models show approximately linear relationships (Figure 1). This was supported by lower AIC and BIC values of the linear models, which represents better model fit (Table S6). We used the same hemoglobin measurement for the continuous exposure as we did for the categorical exposure. After adjusting for potential confounders, each 10 g/L increase in postpartum hemoglobin was associated with 2.99 (95% CI: 2.12–3.86) more meters walked within 6‐min and 40% lower odds of post‐walk breathlessness (adjusted odds ratio [aOR] = 0.60, 95% CI: 0.49–0.74) (Table S6). Women who did not participate in the 6‐min walk had worse postpartum anemia (Table S7).

**TABLE 2:** Adjusted association between postpartum anemia and physical capacity, vigor, fatigue (general, physical, emotional, mental), other anemia symptoms, overall fatigue, difficulties breastfeeding, expected difficulties with usual activities, post‐walk breathlessness, illness, and pain.

	Postpartum anemia		
	Severe	Mild/moderate	None
	β‐coefficient (95% CI)		
Physical capacity (6‐min walk)	−3.56 (−7.17–0.04)	0.00	6.37 (3.28–9.46)

	Incident rate ratio (95% CI)		
Vigor	0.99 (0.97–1.01)	1.00	1.00 (0.99–1.02)
General fatigue	1.16 (1.09–1.23)	1.00	1.06 (1.00–1.11)
Physical fatigue	1.16 (1.09–1.23)	1.00	1.08 (1.03–1.13)
Emotional fatigue	1.10 (0.98–1.24)	1.00	1.21 (1.09–1.34)
Mental fatigue	1.18 (1.03–1.36)	1.00	1.18 (1.05–1.36)
Other anemia symptoms	1.21 (1.13–1.29)	1.00	1.06 (1.01–1.12)

	Odds ratio (95% CI)		
Overall fatigue	1.22 (1.04–1.42)	1.00	1.17 (1.02–1.34)
Difficulties with breastfeeding	0.95 (0.75–1.21)	1.00	1.00 (0.83–1.22)
Expected difficulties with usual activities	1.48 (1.14–1.91)	1.00	0.97 (0.73–1.27)
Post‐walk breathlessness	2.28 (1.25–4.18)	1.00	0.25 (0.06–1.05)
Illness	0.98 (0.63–1.53)	1.00	0.58 (0.35–0.96)
Pain	0.75 (0.45–1.26)	1.00	1.11 (0.70–1.76)

*Note*: All models included a random effect for hospital and adjust for time to outcome assessment, pre‐birth anemia severity, age, gestational age, multiple gestation, primiparity, placental abnormalities, long‐term infection, hypertensive disorder, labor induction, labor augmentation, pain relief, instrumental birth, episiotomy, trauma to birth canal, labor duration, estimated total blood loss (quadratic term), clinical diagnosis of postpartum hemorrhage, postpartum intravenous fluids, postpartum blood transfusion, small vulnerable newborn, and perinatal death before discharge.

Abbreviation: CI, confidence interval.

**FIGURE 1:**
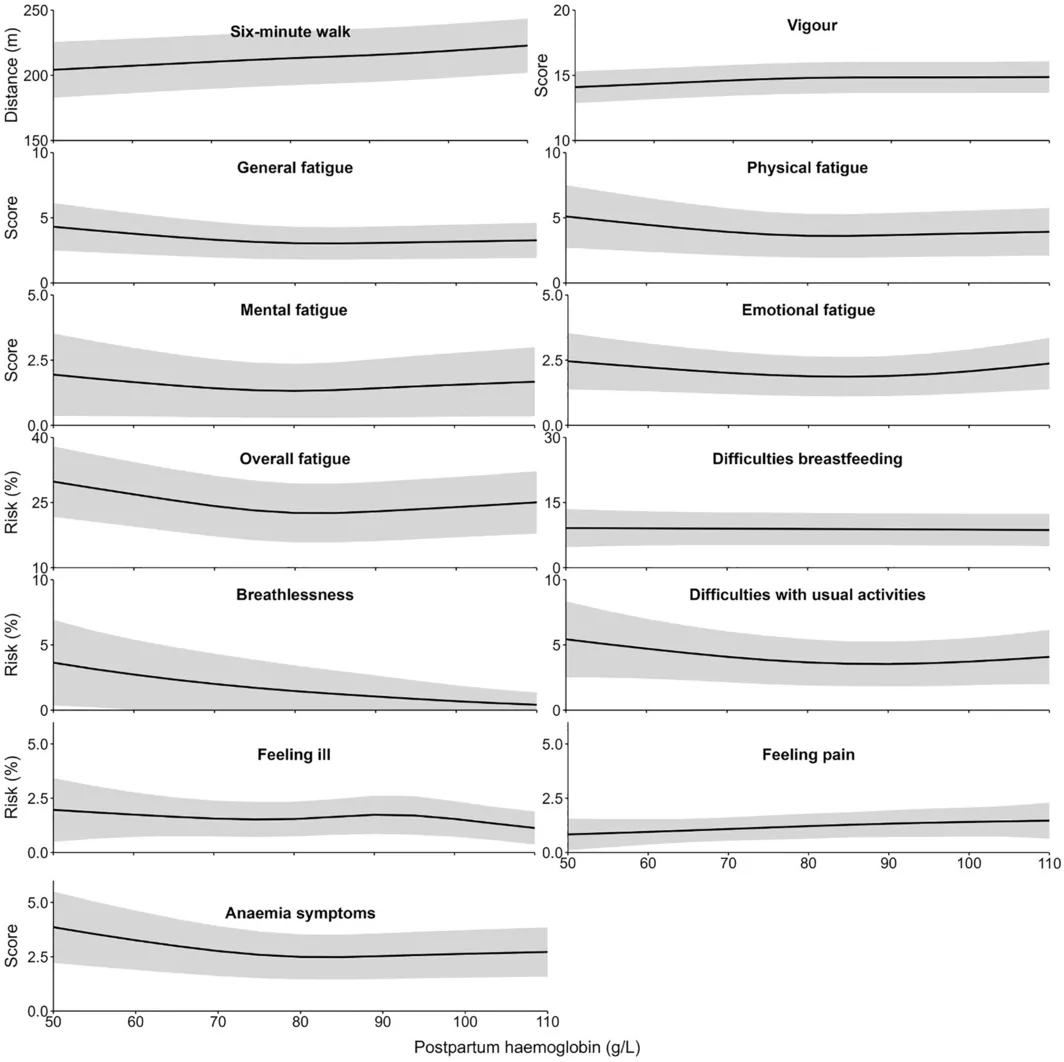
Associations between postpartum hemoglobin concentration (g/L) and physical capacity (6‐min walk), vigor, fatigue (general, physical, emotional, mental), overall fatigue, difficulties breastfeeding, post‐walk breathlessness, expected difficulties with usual activities, illness, pain, and anemia symptoms. Graphs of model‐based predictions from linear regression (physical capacity), negative binomial regression (vigor, general, physical, emotional, and mental fatigue, other anemia symptoms), and logistic regression (overall fatigue, difficulties breastfeeding, post‐walk breathlessness, expected difficulties with usual activities, illness, and pain) models treating the exposure (postpartum hemoglobin concentration (g/L)) as a restricted cubic spline with four knots. Models were adjusted for time to outcome assessment, pre‐birth anemia severity, age, gestational age, multiple gestation, primiparity, placental abnormalities, long‐term infection, hypertensive disorder, labor induction, labor augmentation, pain relief, instrumental birth, episiotomy, trauma to birth canal, labor duration, estimated total blood loss (quadratic term), clinical diagnosis of postpartum hemorrhage, postpartum intravenous fluids, postpartum blood transfusion, small vulnerable newborn, and perinatal death before discharge.

Compared to mild/moderate postpartum anemia, severe anemia was associated with expected difficulties with usual activities (aOR = 1.48, 95% CI: 1.14–1.91), overall fatigue (aOR = 1.22, 95% CI: 1.04–1.42), other anemia symptoms (adjusted incident rate ratio [aIRR] = 1.21, 95% CI: 1.13–1.29), mental fatigue (aIRR = 1.18, 95% CI: 1.03–1.36), general fatigue (aIRR = 1.16, 95% CI: 1.09–1.23), physical fatigue (aIRR = 1.16, 95% CI: 1.09–1.23), and emotional fatigue (aIRR = 1.10, 95% CI: 0.98–1.24) (Table 2). Compared to mild/moderate postpartum anemia, no anemia was associated with less reported illness (aOR = 0.58, 95% CI: 0.35–0.96) and greater emotional fatigue (aIRR = 1.21, 95% CI: 1.09–1.34), mental fatigue (aIRR = 1.18, 95% CI: 1.05–1.36), physical fatigue (aIRR = 1.08, 95% CI: 1.03–1.13), other anemia symptoms (aIRR = 1.06, 95% CI: 1.01–1.12), general fatigue (aIRR = 1.06, 95% CI: 1.00–1.11), and overall fatigue (aOR = 1.17, 95% CI: 1.02–1.34). These non‐linear relationships are shown in the cubic spline models (Figure 1). We found no evidence that postpartum anemia was associated with vigor, breastfeeding difficulties, or pain (Table 2).

We observed similar results when we modeled the score measures as binary outcomes using logistic regression (Tables S8 and S9) and when we removed time at outcome assessment from our multivariable models (Table S10). The effect estimates mostly moved towards the null after we excluded women who received a blood transfusion from the analyses (Table S11).

## 4 DISCUSSION

Lower postpartum hemoglobin was associated with worse physical capacity of new mothers. Women with severe postpartum anemia reported worse wellbeing outcomes compared to women with mild/moderate postpartum anemia, while women without postpartum anemia reported mixed outcomes.

We explored the impact of postpartum anemia on maternal wellbeing in a large cohort of women giving birth with anemia in Nigeria, Tanzania, Pakistan, and Zambia. We were able to examine the impact of severe postpartum anemia on maternal wellbeing due to its high incidence in the study population. However, we lacked information on the causes of anemia. We assessed a range of maternal wellbeing outcomes that women highlighted as important during the postpartum period.^18^ These measures were developed for, and validated in, women with postpartum anemia.^18^ While our primary outcome of physical capacity (measured with a 6‐min walk test) could be impacted by multiple issues, we were able to adjust for important factors like episiotomy and instrumental birth. We measured postpartum hemoglobin with the HemoCue Hb 201+. While this has high accuracy compared with laboratory measurements, some measurement error is expected.^19^ Women who did not take part in the 6‐min walk had worse postpartum anemia, so we may have underestimated its effect on physical capacity. The patient‐reported outcomes were measured at discharge, which may have led to measurement error if women hurried the questionnaire to return home. We explored linear and non‐linear relationships with spline models and modeled them appropriately for each outcome. While there are no established thresholds for postpartum anemia, we used British Society of Hematology cutoffs that broadly match the non‐linear relationships shown in the spline models.^16^, ^17^ We adjusted for a range of potential confounders, including pre‐birth anemia severity and postpartum bleeding. However, our results could still be biased by residual confounding (e.g., by women's mental health status before pregnancy and birth). Our findings were robust to multiple sensitivity analyses.

Postpartum hemoglobin was associated with increased physical capacity and reduced breathlessness. This is in line with randomized trials that show increasing hemoglobin improves physical capacity.^7^, ^20^ Lower hemoglobin may result in less oxygen delivery to the tissues, which would impact cellular energy production.^4^, ^5^ Cardiac and skeletal muscles have high energy demands.

Severe postpartum anemia was associated with a range of adverse maternal wellbeing outcomes when compared to mild/moderate anemia. This may result from compensatory mechanisms that maintain an adequate oxygen supply to the tissues in mild/moderate anemia but fail at lower hemoglobin concentrations.^21^ While (to our knowledge) this is the first study to assess the impact of severe anemia in the postpartum period, there is extensive evidence of its adverse impact on pregnant women.^9^, ^22^ Severe postpartum anemia is an important condition for women who give birth with anemia and/or bleed heavily during childbirth (e.g., cesarean births).^23^

Although the study was restricted to women with moderate or severe anemia before birth, 14% of women were not anemic after birth. While this seems paradoxical, this was likely due to a combination of blood transfusion and/or hemoconcentration. Women who were not anemic after birth had the highest hemoglobin on average before birth. It is possible their true pre‐birth hemoglobin was ≥100 g/L and they were misclassified as having hemoglobin <100 g/L before birth due to measurement error or regression towards the mean. Compared to mild/moderate postpartum anemia, no postpartum anemia was associated with less reported illness but greater emotional and mental fatigue. This conflicts with randomized trials that show treating anemia reduces postpartum depression and fatigue.^6^, ^8^ These associations were reduced when we excluded women who received a blood transfusion from the analysis, which suggests they were driven by the experience of transfusion. Our results could also be confounded by pre‐birth blood transfusion and/or peripartum iron supplementation, which we were unable to adjust for. Differential misclassification may also have occurred. Women who had mild/moderate postpartum anemia were more anemic before birth, so could have normalized fatigue and reported their symptoms less. We recommend interpreting these U‐shaped relationships with caution.

We found no evidence that postpartum anemia was associated with vigor, breastfeeding difficulties, or pain. These outcomes have diverse causes that may have impacted women's wellbeing more than their anemia status. For example, breastfeeding difficulties can occur because of the baby's status as well as those of the mother.

Future studies could examine the impact of postpartum anemia on maternal wellbeing in women who are not anemic before birth. Hemoglobin was measured around 12 h after birth. While this is in line with guidelines for women with antepartum anemia, hemoglobin is highly variable in this period.^16^ Maternal wellbeing was measured before discharge from hospital. Future studies could examine the effect of postpartum anemia on long‐term outcomes like postpartum depression and mother‐infant bonding, measuring hemoglobin after 48‐h postpartum.

## 5 CONCLUSION

Low postpartum hemoglobin was associated with adverse wellbeing in new mothers. While we cannot infer causation from this observational study, this finding is in line with randomized controlled trials that show treating anemia improves a range of wellbeing outcomes.^7^, ^8^, ^20^ Our results support recommendations to prevent and correct low postpartum hemoglobin.^16^, ^24^

## AUTHOR CONTRIBUTIONS

JL: Conceptualization, methodology, formal statistical analysis, visualization, and writing of the original draft. IR: Conceptualization, methodology, supervision, funding acquisition, writing of the original draft and draft revisions. HSS: Funding acquisition and review and editing of the manuscript. KK did the writing of the original draft and draft revisions. FAB, RC, KJ, AK, MKL, PM, OO, and BV: Data collection, data interpretation, and review and editing of the manuscript. EB and MA: Project administration and review and editing of the manuscript. AG and DP: Data curation and review and editing of the manuscript. PM: Methodology and review and editing of the manuscript. AB and RM: Review and editing of the manuscript. JL, AG, DP, and RM: Accessed and verified the data. All authors are responsible for the decision to submit the manuscript.

## FUNDING INFORMATION

The WOMAN‐2 trial was funded by Wellcome (WT208870/Z/17/Z) and the Bill & Melinda Gates Foundation (INV‐007787).

## CONFLICT OF INTEREST STATEMENT

No competing interests were disclosed.

## AI DISCLOSURE

ChatGPT (https://chat.openai.com/) was used to reword short phrases.

## Supporting information


**Table S1.** Patient‐reported outcome questionnaire and corresponding maternal wellbeing outcomes.
**Figure S1.** Directed acyclic graph showing the potential causal pathways between postpartum anemia and postpartum wellbeing.
**Table S2.** Description of covariates included in the regression models.
**Table S3.** Participants’ characteristics, by postpartum anemia status.
**Table S4.** Distribution of maternal wellbeing outcome measures.
**Table S5.** Unadjusted association between postpartum anemia and physical capacity, vigor, fatigue (general, physical, emotional, mental), other anemia symptoms, overall fatigue, difficulties breastfeeding, expected difficulties with usual activities, post‐walk breathlessness, illness, and pain.
**Table S6.** Adjusted association between postpartum hemoglobin and physical capacity and post‐walk breathlessness (modeled as categorical and continuous exposures).
**Table S7.** Participants’ characteristics, by participation in the six‐minute walk test.
**Table S8.** Unadjusted association between postpartum anemia and vigor, fatigue (general, physical, emotional, mental), and other anemia symptoms (sensitivity analysis using logistic regression to model count measures as binary outcomes).
**Table S9.** Adjusted association between postpartum anemia and vigor, fatigue (general, physical, emotional, mental), and other anemia symptoms (sensitivity analysis using logistic regression to model count measures as binary outcomes).
**Table S10.** Adjusted association between postpartum anemia and physical capacity, vigor, fatigue (general, physical, emotional, mental), other anemia symptoms, overall fatigue, difficulties breastfeeding, expected difficulties with usual activities, post‐walk breathlessness, illness, and pain (sensitivity analysis that does not adjust for time at outcome assessment).
**Table S11.** Adjusted association between postpartum anemia and physical capacity, vigor, fatigue (general, physical, emotional, mental), other anemia symptoms, overall fatigue, difficulties breastfeeding, expected difficulties with usual activities, post‐walk breathlessness, illness, and pain (sensitivity analysis that excludes women who received a blood transfusion).

## Data Availability

Individual deidentified WOMAN‐2 trial patient data, including a data dictionary, will be made available via our data sharing portal, The Free Bank of Injury and Emergency Research Data (freebird.lshtm.ac.uk) website.

## APPENDIX A THE WOMAN‐2 TRIAL COLLABORATORS

Writing Committee: Judith Lieber (Chair). Clinical Trials Unit, London School of Hygiene & Tropical Medicine, London, UK; Katharine Ker. Clinical Trials Unit, London School of Hygiene & Tropical Medicine, London, UK; Folasade Adenike Bello*. Department of Obstetrics and Gynecology, University of Ibadan College of Medicine, Ibadan, Nigeria; Rizwana Chaudhri*. Global Institute of Human Development, Shifa Tameer‐e‐Millat University, Islamabad, Pakistan; Kiran Javaid. Global Institute of Human Development, Shifa Tameer‐e‐Millat University, Islamabad, Pakistan; Aasia Kayani. Global Institute of Human Development, Shifa Tameer‐e‐Millat University, Islamabad, Pakistan; Mwansa Ketty Lubeya. University of Zambia School of Medicine, Lusaka, Zambia; Projestine Muganyizi*. Muhimbili University of Health and Allied Sciences & University of Dar es Salaam Mbeya College of Health and Allied Sciences, Dar es Salaam, Tanzania; Oladapo Olayemi*. Department of Obstetrics and Gynecology, University of Ibadan College of Medicine, Ibadan, Nigeria UK; Bellington Vwalika*. University of Zambia School of Medicine, Lusaka, Zambia; Monica Arribas. Clinical Trials Unit, London School of Hygiene & Tropical Medicine, London, UK; Eni Balogun. Clinical Trials Unit, London School of Hygiene & Tropical Medicine, London, UK; Amy Brenner. Clinical Trials Unit, London School of Hygiene & Tropical Medicine, London, UK; Amber Geer. Clinical Trials Unit, London School of Hygiene & Tropical Medicine, London, UK; Poppy Mallinson. Department of Non‐Communicable Disease Epidemiology, London School of Hygiene & Tropical Medicine, London, UK; Raoul Mansukhani. Clinical Trials Unit, London School of Hygiene & Tropical Medicine, London, UK; Danielle Prowse. Clinical Trials Unit, London School of Hygiene & Tropical Medicine, London, UK; Haleema Shakur‐Still*. Clinical Trials Unit, London School of Hygiene & Tropical Medicine, London; Ian Roberts*. Clinical Trials Unit, London School of Hygiene & Tropical Medicine, London, UK.

*denotes Professors.

WOMAN‐2 Trial steering committee: France Donnay (Chair), Racheal Phiri (patient representative), Tina Lavender, Ghazala Mahmud, Haleema Shakur‐Still, Ian Roberts, Jens Kieckbusch (Observer), James Lawrence (Observer), Sari Watson (Observer).

Data monitoring committee: Jane Armitage (Chair), Maria Quigley, Olufemi T. Oladapo.

Protocol committee: Sabaratnam Arulkumaran, Imelda Bates, Rizwana Chaudhri, Bukola Fawole, Katharine Ker, Ian Roberts, Haleema Shakur‐Still.

Central Coordinating Team (Clinical Trials Unit, London School of Hygiene and Tropical Medicine): Eni Balogun (senior trial manager/research fellow), Danielle Prowse (senior data manager), Danielle Beaumont (senior trial manager/research fellow), Charlotte Fleming (assistant trial manager—September 2018—January 2022), Monica Arribas (senior trial manager/research fellow), Collette Barrow (trial administrator), Llion Roberts (assistant trial manager—April 2022–), Amber Geer (data manager), Julio Gil Onandia (trial assistant), Madeleine Cargill (data assistant September 2018–September 2020) Andrew Thayne (assistant data manager), Christiana Alao (data assistant—January 2022–), Petula Buck (trial assistant), Raoul Mansukhani, (statistician), Tim Collier (statistician), Katharine Ker (assistant professor), Mbwana Mohammed (IT systems manager), Amy Brenner (assistant professor), Myriam Benyahia (project coordinator), Judith Lieber, Haleema Shakur‐Still (chief investigator), Ian Roberts (chief investigator).

Pakistan coordinating team (Global Institute of Human Development, Shifa Tameer‐e‐Millat University, Islamabad, Pakistan): Rizwana Chaudhri (coordinating centre director), Aasia Kayani (research coordinator), Kiran Javaid (assistant research coordinator).

Tanzania coordinating team (Muhimbili University of Health and Allied Sciences, Dar es Salaam, Tanzania): Projestine Muganyizi (coordinating centre director), Alice Kawala (assistant trials coordinator), Rose Temba (assistant trials coordinator).

Nigeria coordinating team (College of Medicine, University of Ibadan, Nigeria): Oladapo Olayemi (coordinating centre director), Folasade Adenike Bello (coordinating centre director), Olujide Okunade (trials coordinator).

Zambia National coordinator: Bellington Vwalika (University Teaching Hospital, Lusaka, Zambia).

Sites

Pakistan (11 025 participants)—Ayub Teaching Hospital units A, B and C (1391), Aziz‐un‐Nisa Abbasi, Ruqqia Sultana, Shehla Noor, Ansa Islam, Sadia Bibi, Sundus Khan, Wajeeha Khurshid; Chandka SMBBMU Shaikh Zaid Women Hospital Unit 1 (1145), Shahida Magsi, Basma Zia; Jinnah Postgraduate Medical Centre Unit 1/ward 8 and Unit 2/ward 9 (856), Haleema Yasmin, Khadija Bano, Saba Khan; Bahawal Victoria Hospital Bahawalpur Units 1 & 2 (826), Naheed Fatima, Bushra Sher Zaman, Sohail Mahmood Chaudhry, Shakila Yasmin, Khiaynat Sarwar Hashmi, Nadia Babar; Jinnah Hospital Lahore (727), Arif Tajammul, Alia Bashir, Farah Siddique; Civil Hospital Karachi Units 1 and 3 (699), Fouzia Perveen, Nusrat Shah Kamal, Riffat Jaleel, Sarah Kazi, Aruna Hira, Pushpa Makhija; MCH PIMS Unit 1 and 2 (681), Syeda Batool Mazhar, Nasira Tasnim, Saera Afghan, Mussarat Batool, Sobia Luqman; Chandka SMBBMU Shaikh Zaid Women Hospital Unit 2 and 3 (673), Fouzia Kashif, Shaista Hifaz Abro, Javeria Abro; Bolan Medical Complex Unit 3 and 4 (666), Najma Ghaffar, Rakhshanda Mushtaq, Safia Bibi; Sir Ganga Ram Hospital Units 1–4 (595), Shamsa Humayun, Zohra Khanum, Noreen Akmal, Shamila Ijaz Munir, Aisha Malik, Amna Zia Eusaph, Sofia Manzoor, Khola Yousaf, Misbah Kausar, Yasmeen Waqas; Bolan Medical Complex (Sandeman Provincial Hospital) Units 1 and 2 (576), Naila Ehsan, Aisha Siddiqa, Uzma Suhail Afridi, Nosheen Sikandar, Rehana Kamaal; Nishtar Hospital Unit 2 (310), Mehnaz Khakwani, Rashida Perveen; Nishtar Hospital Unit 1 (305), Huma Quddusi, Hajira Masood, Syeda Ali, Asma Akhter; Koohi Goth Hospital Karachi (276), Mubashra Samina, Sana Ishfaque; Holy Family Hospital Unit 1 (264), Rizwana Chaudhri, Humaira Bilqis; Nishtar Hospital Unit 3 (212), Shahid Irshad Rao, Saima Ashraf, Uzma Shaheen; Services Hospital Unit 2 (121), Tayyiba Wasim, Natasha Rasool; Holy Family Hospital Unit 2 (152), Nabeela Waheed, Khansa Iqbal; Aziz Bhatti Shaheed Teaching Hospital (132), Shahida Husain Tarar, Rizwana Khalid; Services Hospital Unit 1 (121), Rubina Sohail, Shehla Kanwal; Benazir Bhutto Shaheed Hospital (121), Shagufta Saeed Sial, Humera Noreen; Federal Government Polyclinic (88), Naila Israr; Military Hospital Rawalpindi (34), Shehla Baqai, Asifa Siraj.

Tanzania (2029 participants)—Mwananyamala Regional Referral Hospital (730), Luzango Maembe, Julius Nyambarino; Dodoma Regional Referral Hospital (393), Enid Simon Chiwanga, Lazaro Y. Madeha; Mount Meru Regional Referral Hospital (278), Francis Ngimwichi Joseph, Evelyne Ludovick; Amana Hospital (179), Baya H Kissiwa, Sangalala Mtingele; Muhimbili National Hospital (169), Vincent Tarimo, Alfred Secha; Temeke Hospital (155), Hudson August Manyanga, Idrisa Hussein; Tumbi Hospital (85), Alphonce Moyo, Delila Msigwa; Mbeya Zonal Referral Hospital (40), France John Rwegoshora, Delfina Mkenda.

Nigeria (1326 participants)—Ilorin General Hospital (350), Mojisola Mobolaji‐Ojibara, Michael Babalola; Mother & Child Hospital Akure (250), Owa Olorunfemi Oludele, Samuel Ojo; Adeoyo Maternity Hospital (242), Oladipo Aremu, Kehinde Ogundairo; State Hospital Oyo (230), Oyewole Tunde Aremu, Gazaal Rasheedat; Muhammad Abdullahi Wase Specialist Hospital Kano (121), Iman Usman, Auwal Shuaibu; Ladoke Akintola University of Technology Teaching Hospital Ogbomoso (88), Adewale Adeyemi, Olufemi Aworinde; University of Medical Sciences Teaching Hospital Akure (41), Theresa Irinyenikan.

Zambia (688 participants)—Women and Newborn Hospital (668), Mwansa Ketty Lubeya, Kenneth Chanda.
